# First clinical experience with the Transthyretin Amyloidosis-Quality of Life questionnaire (ATTR-QoL): associations with clinical characteristics and established patient-reported outcomes

**DOI:** 10.1093/ehjqcco/qcag015

**Published:** 2026-02-12

**Authors:** Dimitrios Bampatsias, Sergio Teruya, Alfonsina Mirabal Santos, Juliana C Levy, Kristen Hsu, Phaedra T Johnson, Sabrina Rebello, Andrew Lovley, Kaitlin LaGasse, Kristen L McCausland, Karan Wats, Mathew S Maurer

**Affiliations:** Division of Cardiology, Cardiac Amyloidosis Program, Columbia University Irving Medical Center, New York Presbyterian Hospital, 622 West 168th Street, PH12 Stem Room 134, New York, NY 10032, USA; Division of Cardiology, Cardiac Amyloidosis Program, Columbia University Irving Medical Center, New York Presbyterian Hospital, 622 West 168th Street, PH12 Stem Room 134, New York, NY 10032, USA; Division of Cardiology, Cardiac Amyloidosis Program, Columbia University Irving Medical Center, New York Presbyterian Hospital, 622 West 168th Street, PH12 Stem Room 134, New York, NY 10032, USA; Division of Cardiology, Cardiac Amyloidosis Program, Columbia University Irving Medical Center, New York Presbyterian Hospital, 622 West 168th Street, PH12 Stem Room 134, New York, NY 10032, USA; Amyloidosis Research Consortium, Newton, MA 02460, USA; Amyloidosis Research Consortium, Newton, MA 02460, USA; Amyloidosis Research Consortium, Newton, MA 02460, USA; IQVIA Quality Metric, Inc., Providence, RI 02919, USA; IQVIA Quality Metric, Inc., Providence, RI 02919, USA; IQVIA Quality Metric, Inc., Providence, RI 02919, USA; Division of Cardiology, Cardiac Amyloidosis Program, Columbia University Irving Medical Center, New York Presbyterian Hospital, 622 West 168th Street, PH12 Stem Room 134, New York, NY 10032, USA; Division of Cardiology, Cardiac Amyloidosis Program, Columbia University Irving Medical Center, New York Presbyterian Hospital, 622 West 168th Street, PH12 Stem Room 134, New York, NY 10032, USA

**Keywords:** Transthyretin amyloidosis, Quality of life, PRO, ATTR-QoL

## Abstract

**Aims:**

Transthyretin amyloidosis (ATTR) is a multisystemic disease impacting quality of life (QOL) through various symptoms. The Transthyretin Amyloidosis—Quality of Life Questionnaire (ATTR-QOL) was developed to assess disease-specific effects on QOL, but its associations with established patient-reported outcomes measures (PROs), clinical markers, and functional tests remain unexplored.

**Methods and results:**

This cross-sectional study included consecutive ATTR patients at Columbia University between 3/2024 and 1/2025 who completed the ATTR-QOL. The questionnaire includes 5 symptom and 4 impact scores (range: 0–100, higher scores indicate worse status). Clinical parameters, PROs, and functionality were recorded at the same visit. PROs of interest included the Kansas City Cardiomyopathy Questionnaire (KCCQ), SF36v2 and Composite Autonomic Symptom Score-31 (COMPASS-31), while 6-minute walk test and Short Physical Performance Battery Test were recorded as functionality measures. 194 patients (137 cardiomyopathy, 48 mixed, 9 neuropathy) were included. Most had early-stage disease [National Amyloidosis Centre (NAC) Stage 1: 70%, Stage 2: 22%, Stage 3: 8%], and all were on disease-modifying treatment. ATTR-QOL exhibited good convergent validity with clinical parameters, functionality testing, and established QOL tools. Additionally, ATTR-QOL demonstrated strong known-groups discriminant validity, as evidenced by its ability to differentiate between various clinical and functional groups, as well as established patient-reported outcome (PRO) categories.

**Conclusion:**

ATTR-QOL, as a disease-specific PRO, effectively captures the multidimensional impact of ATTR and is associated with clinical parameters and functionality in patients with ATTR.

## Graphical Abstract

**Graphical Abstract:**
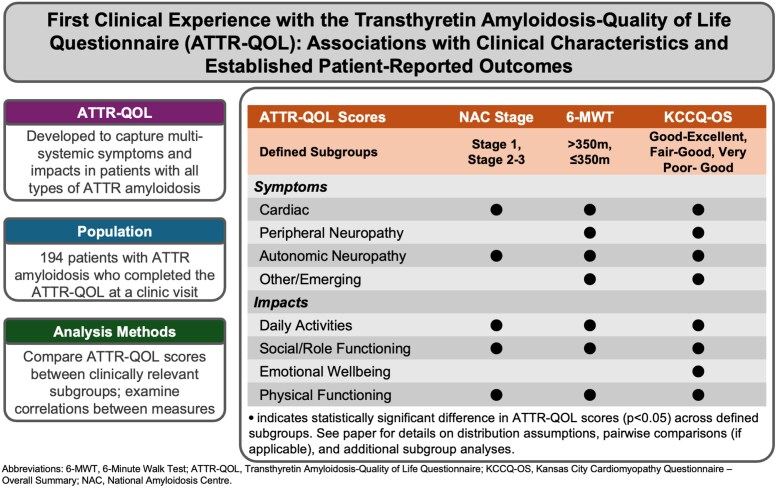

Key Learning Points What is already known:

- ATTR is a multisystemic disease impacting quality of life (QOL) through various symptoms.
- Organ specific or general health Patient Reported Outcomes (PROs) measures have been used in ATTR, but do not capture disease burden in a holistic and unified way.
- Transthyretin Amyloidosis-Quality of Life Questionnaire (ATTR-QOL) can effectively capture disease burden, but has not been studied in clinical settings.

What this study adds:

- ATTR-QOL: is applicable in clinical settings.
- ATTR-QOL exhibits good convergent validity with clinical parameters, functionality, and QOL.
- ATTR-QOL demonstrates known-groups discriminant validity.

## Introduction

Transthyretin amyloidosis (ATTR) is a progressive disease caused by misfolded transthyretin deposits, affecting multiple organs. It occurs as a genetic form (ATTRv) with over 130 known mutations or as a non-genetic, age-related form (ATTRwt).^1,2^ ATTRwt primarily leads to cardiomyopathy (ATTR-CM) but also involves neuropathic, autonomic, and musculoskeletal symptoms.^1-3^ ATTRv varies in presentation, causing neuropathy (ATTR-PN), cardiomyopathy, or a mixed phenotype depending on the mutation.^1-3^ Regardless of type, ATTR results in a wide range of symptoms impacting daily life, healthcare utilization, and overall health-related quality of life (HR-QOL).^4-6^ Impaired QOL and its progessive worsening, as captured by patient-reported outcome (PRO) measures, has been associated with worse outcomes and increased healtcare costs in other cardiovascular conditions.^7-9^

Until recently, there was a lack of an ATTR-specific PRO measure, which is required for a consistent and comprehensive assessment of the different aspects of HR-QOL in ATTR.^10^ A combination of PROs developed for different conditions and general health status [Norfolk-QOL-Diabetic Neuropathy, Composite Autonomic Symptom Score-31 (COMPASS-31), Kansas City Cardiomyopathy Questionnaire (KCCQ), SF-36v2] have been used in clinical and research settings to evaluate symptom severity and impact on HR-QOL, failing to capture the burden of ATTR in a holistic and disease-specific way.^10^ To address this issue, the Amyloidosis Research Consortium (ARC) recently developed the Transthyretin Amyloidosis—Quality of Life Questionnaire (ATTR-QOL), the first ATTR-specific PRO.^10^ The ATTR-QOL survey items evaluate the symptoms and their impact on HR-QOL as follows: (i) Frequency and severity of cardiac, peripheral neuropathic, autonomic and other/emerging symptoms, (ii) Impact of symptoms on daily activities, social/role functioning, emotional well-being and physical functioning.

Initial validation of the measure has been conducted, which confirms that the ATTR-QOL effectively captures the different aspects of the disease.^11,12^ It denotes the magnitude of cardiac, neuropathic and other symptoms, as well as daily impact on ATTR patients with different genotypes. Notably, patients who were V122I carriers had higher symptom and impact scores.^11^ However, the use and applicability of the ATTR-QOL in clinical settings has remained untested to date, as initial analyses of the reliability and validity of the measure utilized self-reported data from online voluntary patient surveys, which did not include clinical parameters.^12^ Associations with clinical parameters and its ability to distinguish disease severity stages need to be studied. Additionally, given the importance of functional capacity in ATTR, linking ATTR-QOL with established measures like the 6-minute walk test (6-MWT) and Short Physical Performance Battery (SPPB) could enhance its applicability.^13^ The aim of this study is to explore the association between ATTR-QOL scores and clinical characteristics, functionality tests, and other PROs in patients with ATTR in a real-world clinical setting.

## Methodology

The present study is a cross-sectional study including consecutive patients with ATTR being followed at the Cardiac Amyloidosis Program in Columbia University Medical Centre from March 2024 to January 2025. All patients had an established diagnosis of ATTR, either biopsy proven or by non-biopsy approach, based on standard clinical criteria.^13^ Study participants were asked to complete the questionnaires and functionality tests the same day as their visit. In a few cases when the patients were unable to complete the questionnaires during the visit, the forms were emailed to the participants and completed at home, within a week following the visit. All forms were answered electronically.

As part of the clinical assessment, we recorded past medical history, concomitant medication, biomarkers, heart failure severity based on New York Heart Class (NYHA) and disease staging based on National Amyloidosis Centre (NAC) and Columbia Score. The Columbia score for amyloidosis staging was computed from: the NAC stage (0 point for stage 1, 1 for each stage increase) + daily furosemide dose equivalents (0 points: 0 mg/kg, 1 point: between >0 to 0.5 mg/kg, 2 points: > 0.5 to 1 mg/kg, 3 points: > 1 mg/kg) + NYHA functional class (1 point for each class), resulting in a score ranging from 0 to 9.^14^ Based on Columbia score, disease can be characterized as: early phase (1–3 points), intermediate phase (4–6 points), late phase (7–9 points).^14^

Informed consent form was obtained from all subjects before participating in the study and an Institutional Review Board approved the study protocol. The present study adheres to the ‘Strengthening The Reporting of Observational Studies in Epidemiology (STROBE)’ reporting guidelines.^15^

## Instruments

### ATTR-QOL

The ATTR-QOL is a recently developed disease-specific PRO measure created by the ARC.^10-12^ Items assess the frequency and severity of the diverse symptoms of ATTR amyloidosis, as well as their impacts on quality of life for the past 4 weeks. Items from the Symptom domain can be used to derive Cardiac, Peripheral Neuropathy, Autonomic Neuropathy, Gastrointestinal (subscale of Autonomic Neuropathy), and Other/Emerging symptoms sub-domain scores. The Impact domain can be used to derive Daily Activities, Social/Role Functioning, Emotional Well-being, and Physical Functioning sub-domain scores. All scores range from 0 to 100, with higher scores indicating a greater disease burden.

### Kansas city cardiomyopathy 23 item questionnaire (KCCQ-23 item)

The KCCQ is a disease-specific PRO measure of health status for patients with heart failure.^16^ The KCCQ consists of 7 domains with 23 items and a recall period of the last 2 weeks. The five domains quantified are physical limitation, symptom frequency, symptom severity, symptom burden, social limitations, self-efficacy, and quality of life. Scores are scaled from 0 to 100 with higher scores indicating better HR-QOL.^16^

### SF-36v2 health survey

The SF-36v2 health survey (SF-36v2) is a PRO measure of general HR-QOL.^17^ The questionnaire consists of 36 items with a recall period of the past 4 weeks. All except one item are assigned to one of the eight domains covering: physical functioning, physical role functioning, bodily pain, general health, vitality, social functioning, emotional role functioning, and mental health.^17^ Out of the eight subscales, each representing one health domain, two summary measures can be constructed: the physical component summary (PCS) for self-perceived physical health and the mental component summary (MCS) for self-perceived mental health.^17^ Norm-based scores are standardized to represent a mean of 50 and a standard deviation of 10 in the general population with higher scores indicating better HR-QOL.

### Composite autonomic symptom score-31 (COMPASS-31)

The COMPASS-31 is a generic PRO measure of autonomic neuropathy symptoms. The questionnaire includes 31 questions with a recall period of 1 year for most questions. Assessment is through six weighted domains: orthostatic intolerance, vasomotor, secretomotor, gastrointestinal, bladder, and pupillomotor, with higher scores indicating worse autonomic dysfunction.^18^

### EQ-5D-5L

EQ-5D-5L is a PRO measure of health status that has two components: the EQ-5D descriptive system and the EQ visual analogue scale (EQ VAS).^19^ The descriptive system includes five dimensions: mobility, self-care, usual activities, pain/discomfort, and anxiety/depression. Each dimension has five levels: no problems, slight problems, moderate problems, severe problems and extreme problems. The EQ VAS records the patient’s self-rated health on a visual analogue scale where the endpoints are labelled ‘The best health you can imagine’, (scaled as 100) and ‘The worst health you can imagine’ (scaled as 0). The VAS can be used as a quantitative measure of health outcome that reflects the patient’s own judgement.^19^

### Six-minute walk test

The Six-minute walk test (6-MWT) is a performance outcome measure of functional capacity that was administered according to standard guidelines.^20^ A single walk test without practice was administered. Participants were instructed to walk continuously on a 20 m hospital corridor, covering as much ground as they could for 6 min. Encouragement was given every minute in a standardized fashion. Total distance walked in 6 min was recorded in metres. To facilitate interpretation of the results, 6-MWT results were split in the following categories: > 350 m or ≤350 m. The cut-off of 350 m was selected based on previous literature, showing that his cut-off can has independent predictive value in ATTR.^21^

### Short physical performance battery (SPPB)

The SPPB is a series of three tests to assess lower extremity physical function: a 4-m walk at usual pace, time to complete five unassisted chair stands, and three standing balance tests, each held for 10 s, and the stances are progressively more difficult.^21^ Each test is scored on a 0 to 4 scale using previously validated norms and summed for an overall score range of 0 to 12, with 0 indicating the lowest physical performance, and scores of 12 indicating the highest performance. To facilitate interpretation, SPPB scores were split into the following previously defined categories: Low (SPPB score range = 0–6), Moderate (7–9), and High (10–12).^22^

If a patient was unfit to perform the functionality testing, the lowest possible value (0) was recorded in the dataset.

## Statistical analysis

The Shapiro–Wilk test and histogram inspection were used to assess ATTR-QOL data distribution. Continuous variables are expressed as mean [standard deviation (SD)] if normally distributed or median [interquartile range (IQR)] if not; categorical variables are expressed as frequency (percentage). Student's *t*-test or Mann–Whitney U-test was used to determine statistical significance for normally distributed and non-normally distributed continuous variables with two groups, respectively. For variables with >2 groups, analysis of variance (ANOVA) or Kruskal Wallis test were used. Spearman’s coefficient was used to examine the correlation between continuous variables. All tests were two-sided (where applicable) and statistical significance was defined as *P* < 0.05. All analyses were adjusted for multiple comparisons using the False Discovery Rate (FDR) method. All analyses were performed using SAS Studio v3.81 and SAS v9.4 (SAS Institute, Cary, NC, USA) and R Statistical Software, version 4.2.1 (R Foundation for Statistical Computing, Vienna, Austria).

## Results

### Total cohort

A total of 194 patients (86% male, 77% White) with a median age of 80 years (44–100) were included in the present study (*Table 1*). One-hundred forty (*N* = 140) patients were seen at the same time but did not complete the questionnaire. There were no significant differences in terms of age, genotype and disease severity between included and excluded patients (see Supplementary material online, *Table S1*). In the present study, most patients had ATTR wild-type (*N* = 148, 76%), while the most common variants were V122I (*N* = 18, 9%) and Thr60Ala (*N* = 8, 4%). Regarding phenotype, 137 patients had a predominantly cardiac phenotype (70%), 48 had a mixed phenotype (25%), and 9 had a predominantly neuropathic phenotype (5%). All patients were receiving disease-modifying therapy (81% stabilizer, 4.6% silencer, 14.4% combination therapy). In terms of disease severity, most patients had early-stage disease based on NAC or Columbia staging, with relatively low cardiac biomarker levels. Regarding functionality, the present cohort had relatively preserved functionality with a median 6-MWT distance of 386 m (299–467) and 61% of patients having 6-MWT >350 m, and 65% of patients having SPPPB >10. Quality of life data indicated a median KCCQ-OS score of 83 (64–96), suggestive of relatively preserved quality of life. ATTR-QOL scores are shown in Supplementary material online, *Table S2*.

**Table 1:** Demographics and baseline characteristics of the study cohort

N of participants = 194		
Variable	Statistics	
**Age, years**		
**Median, Range**	80	44–100
**Age at Diagnosis, years**		
**Median, Range**	75	39–99
**Time since Diagnosis, years**		
**Median, Range**	3	0–15
**Gender**		
**Male**	166	85.6%
**Race**		
**White or Caucasian**	149	76.8%
**Black or African American**	21	10.8%
**Other**	24	12.4%
**Ethnicity**		
**Hispanic**	7	3.6%
**Employment Status**		
**Employed**	53	27.3%
**Not able to work because of ATTR**	6	3.1%
**Retired**	129	66.5%
**Not employed for other reasons**	6	3.1%
**ATTR Amyloidosis Type**		
**Wild-type ATTR**	148	76.3%
**Hereditary ATTR**	46	23.7%
**Genetic Variant of ATTR Amyloidosis, *N* = 46**		
**Thr60Ala**	8	17.4%
**Val122Ile**	18	39.1%
**Val30Met**	6	13.1%
**Other**	14	30.4%
**Organ Involvement**		
**Cardiac involvement**	187	96.4%
**Peripheral neuropathy**	53	27.3%
**Autonomic neuropathy**	12	6.2%
**Unintended Loss/Gain of 10 lbs. or More in Past 12 Months**		
**Yes**	31	16.0%
**Comorbidities**		
**Atrial fibrillation**	82	42.3%
**Coronary artery disease**	52	26.8%
**Chronic kidney disease**	37	19.1%
**Hypertension**	103	53.1%
**Hyperlipidemia**	110	56.7%
**Diabetes mellitus**	14	7.2%
**Previous stroke or TIA**	19	9.8%
**Carpal tunnel syndrome**	107	55.2%
**Spinal stenosis**	74	38.1%
**NT-proBNP (pg/mL)**		
**Median, Range**	1108	35–**13 450**
**Estimated glomerular filtration rate (eGFR, mL/min/1.73 m^2^)**		
**Median, Range**	60.6	20–127
**High Sensitivity Troponin-T (ng/mL)**		
**Median, Range**	41	0.01–230
**Ejection fraction (%)**		
**Median, Range**	55.00	18–73
**NYHA Class, *N* = 194**		
**1**	52	26.8%
**2**	100	51.6%
**3**	42	21.6%
**4**	0	0.0%
**NAC Stage, *N* = 168**		
**1**	117	69.6%
**2**	37	22.0%
**3**	14	8.3%
**Columbia Disease Stage, *N* = 163**		
**Early**	101	62.0%
**Intermediate**	54	33.1%
**Late**	8	4.9%
**Current ATTR Treatment**		
**Stabilizer treatment**	157	80.9%
**Combination treatment**	28	14.4%
**Silencer treatment**	9	4.6%
**6-Minute Walk Distance (metres), *N* = 140**	368	**20–680**
**6-Minute Walk Distance >350 m**	86	**61%**
**6-Minute Walk Distance ≤350 m**	54	**39%**
**Short Physical Performance Battery Test (SPPB), *N* = 138**	11	4–12
**Higher functionality (SPPB≥10)**	89	65%
**Lower functionality (SPPB < 10)**	49	35%

ATTR, transthyretin amyloidosis; TIA, transient ischaemic attack; NAC, National Amyloidosis Centre; NYHA, New York Heart Association.

### ATTR-QOL and clinical parameters

**Figure 1:**
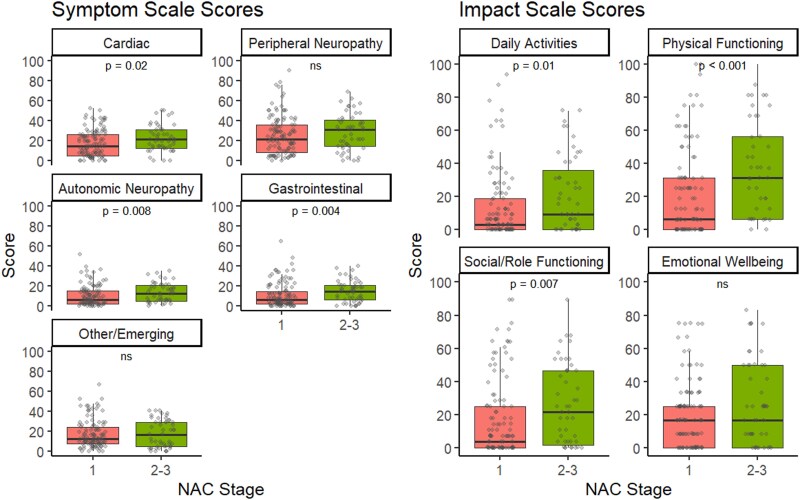
Boxplots of ATTR-QOL symptom and impact scale scores by NAC stage. Higher ATTR-QOL scores indicate a greater disease burden. ATTR-QOL, Transthyretin Amyloidosis—Quality of Life Questionnaire; NAC, National Amyloidosis Centre; ns, non-significant.

ATTR-QOL scores were initially examined across different phenotypes. Patients with a mixed phenotype showed higher ATTR-QOL scores in all Symptom and Impact domains compared with those with a predominantly cardiac or neuropathic phenotype (*Table 2*). ATTR-QOL scores were also evaluated based on disease severity. Patients with higher NYHA class showed significantly worse ATTR-QOL scores, with the greatest numerical differences being observed in the Impact domain (see Supplementary material online, *Figure S1*). Similar findings were observed when ATTR-QOL scores were analyzed by NAC score and Columbia stage, with more advanced disease being associated with higher ATTR-QOL scores (*Figure 1*, Supplementary material online, *Figure S2*). A significant positive correlation was noted between ATTR-QOL Symptom and Impact domain scores, disease staging and biomarkers, except for ‘Other/Emerging Symptoms’ and ‘Emotional Well-being’ (*Table 3*). Overall, ATTR-QOL demonstrated good discriminant validity and correlation with clinical parameters.

**Table 2:** ATTR-QOL scores by ATTR type and affected organs or systems

	ATTR Type						Affected Organs or Systems					
	Wild-type ATTR			Hereditary ATTR			Cardiomyopathy			Mixed Phenotype		
	N	Median	IQR	N	Median	IQR	N	Median	IQR	N	Median	IQR
***ATTR-QOL Symptom Scale Scores***												
**Cardiac**	146	16.7	21.4	46	11.9	21.4	137	14.3	19.0	48	20.2	**17.9**
**Peripheral Neuropathy**	146	21.4	28.6	46	23.8	23.8	137	21.4	26.2	48	33.3	27.4
**Autonomic Neuropathy**	146	6.8	13.6	46	6.1	15.2	137	6.1	13.6	48	10.6	15.9
**Gastrointestinal**	148	8.3	16.7	46	4.2	14.6	139	6.3	16.7	48	12.5	17.7
**Other/Emerging**	148	11.9	21.4	46	9.5	16.7	139	11.9	19.0	48	16.7	22.6
***ATTR-QOL Impact Symptom Scale Scores***												
**Daily Activities**	148	6.3	20.3	46	3.1	28.1	139	6.3	18.8	48	9.4	29.7
**Physical Functioning**	148	12.5	37.5	46	6.3	25.0	139	12.5	37.5	48	12.5	40.6
**Social/Role Functioning**	148	7.1	32.1	46	7.1	25.0	139	7.1	32.1	48	12.5	35.7
**Emotional Well-being**	148	8.3	25.0	46	25.0	33.3	139	8.3	25.0	48	25.0	33.3

ATTR-QOL, Transthyretin Amyloidosis Quality of Life Questionnaire; IQR, interquartile range; SD, standard deviation.

**Table 3:** Spearman correlation coefficients showing the association between biomarkers, functionality tests and patient reported outcomes with ATTR-QOL symptoms and impact scales

		ATTR-QOL Symptom Scales Scores					ATTR-QOL Impact Scales Scores			
Domain	Scale	Cardiac	Peripheral Neuropathy	Autonomic Neuropathy	Gastrointestinal	Other/Emerging	Daily Activities	Physical Functioning	Social/Role Functioning	Emotional Well-being
**Disease staging**	NAC stage	0.18*	0.09	0.21**	0.23**	0.04	0.20*	0.25***	0.24**	0.11
	Columbia Stage	0.22**	0.178	0.23**	0.24**	0.15	0.29***	0.33***	0.32***	0.12
**Biomarkers**	Nt-proBNP (pg/mL)	0.22**	0.15	0.20*	0.21**	0.01	0.30***	0.35***	0.30***	0.1
	hs-Trop T (ng/L)	0.17*	0.22**	0.15	0.16	0.03	0.33***	0.32***	0.34***	0.06
	eGFR (mL/min/1.73 m^2^)	−0.25***	−0.13	−0.21**	−0.22**	−0.09	−0.18*	−0.28***	−0.21**	**−**0.15
**Functionality Tests**	6MWT Distance	−0.24**	−0.38***	−0.38***	−0.36***	−0.24**	−0.57***	−0.55***	−0.40***	**−**0.01
	SPPB Total	−0.25**	−0.34***	−0.28**	−0.29***	−0.22*	−0.53***	−0.47***	−0.34***	**−**0.03
**Patient Reported Outcomes**	KCCQ-PL	−0.51***	−0.61***	−0.55***	−0.55***	−0.40***	−0.71***	−0.69***	−0.66***	**−**0.30***
	KCCQ-SL	−0.60***	−0.58***	−0.50***	−0.49***	−0.37***	−0.64***	−0.55***	−0.70***	**−**0.34***
	KCCQ-QL	−0.71***	−0.54***	−0.58***	−0.57***	−0.43***	−0.49***	−0.44***	−0.62***	**−**0.57***
	KCCQ OS	−0.72***	−0.65***	−0.62***	−0.60***	−0.48***	−0.67***	−0.60***	−0.72***	**−**0.46***
	EQ-5D-5L index	−0.55***	−0.69***	−0.56***	−0.54***	−0.48***	−0.69***	−0.57***	−0.61***	**−**0.44***
	EQ-5D-5L VAS	−0.56***	−0.46***	−0.54***	−0.49***	−0.38***	−0.42***	−0.39***	−0.52***	**−**0.37***
	SF-36v2 PCS	−0.56***	−0.68***	−0.57***	−0.54***	−0.50***	−0.68***	−0.66***	−0.70***	**−**0.35***
	SF-36v2 MCS	−0.49***	−0.34***	−0.45***	−0.44***	−0.39***	−0.33***	−0.19*	−0.43***	**−**0.56***
	COMPASS-31 Score	0.63***	0.56***	0.65***	0.62***	0.58***	0.36***	0.36***	0.51***	0.37***

Asterisks indicate level of statistical significance after false discovery rate correction: *P < 0.05, **P < 0.01, ***P < 0.001.

6MWT, 6-Minute Walk Test; COMPASS-31, Composite Autonomic Symptom Score-31; eGFR, Estimated Glomerular Filtration Rate; Hs-Trop T, High Sensitivity Troponin T; KCCQ-PL, Kansas City Cardiomyopathy Questionnaire Physical Limitations Score; MCS, Mental Component Scale; NAC, National Amyloidosis Center; NT-proBNP, N Terminal Pro B Type Brain Natriuretic Peptide; OS, Overall Score; PCS, Physical Component Scale; QL, Quality of Life; SL, Social Limitation; SPPB, Short Physical Performance Battery Test; VAS, Visual Analogue Scale.

### ATTR-QOL and functionality

**Figure 2:**
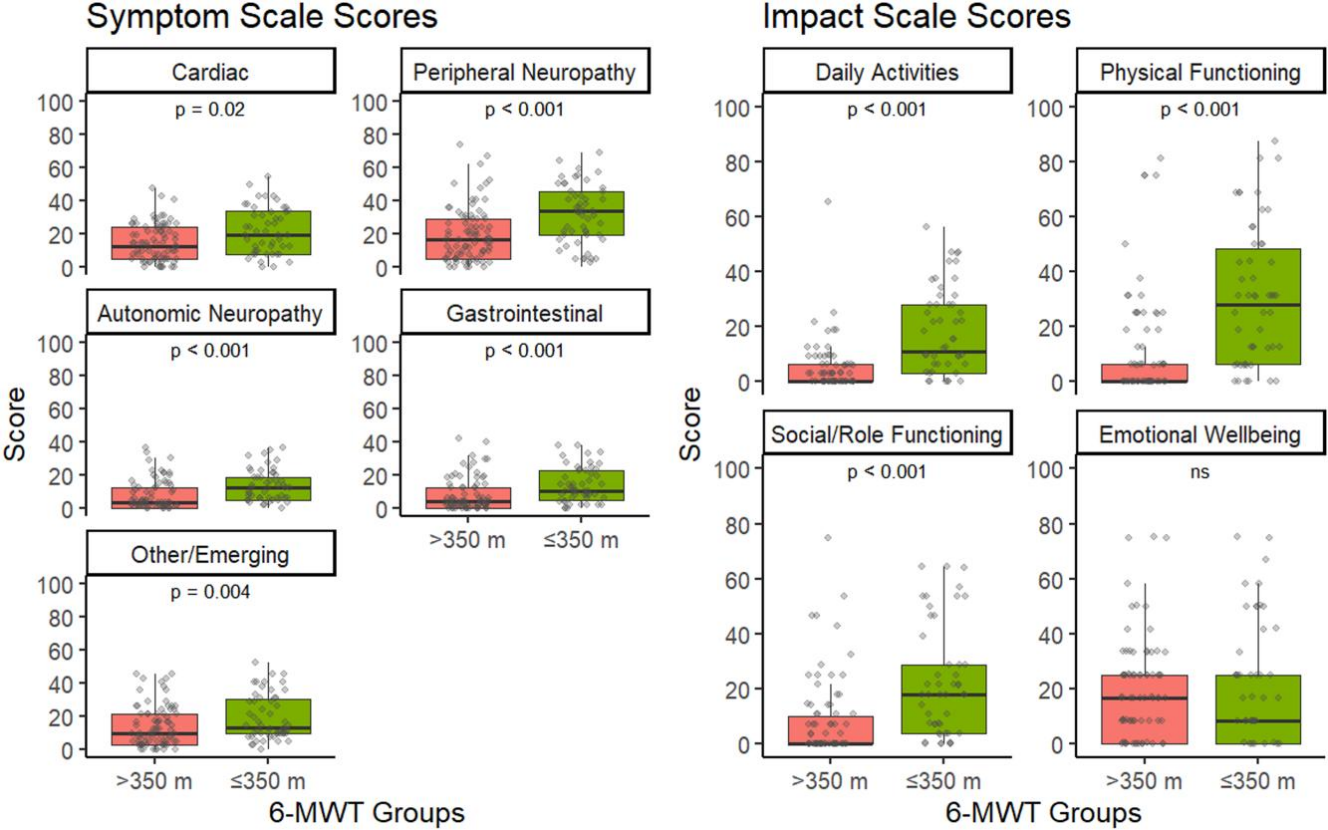
Boxplots of ATTR-QOL symptom and impact scale scores by 6-MWT groups (≤350 m). Higher ATTR-QOL scores indicate a greater disease burden. 6-MWT, 6-minute Walk Test; ATTR-QOL, Transthyretin Amyloidosis—Quality of Life Questionnaire; ns, non-significant.

We next examined ATTR-QOL scores in relation to functional capacity. Continuous ATTR-QOL scores were inversely correlated with both 6-MWT and SPPB scores (*Table 3*). Patients were classified as having higher (> 350 m, *N* = 86) or lower (≤ 350 m, *N* = 54) 6-MWT distance. Patients with lower 6-MWT distance had significantly worse ATTR-QOL Symptoms and Impact domain scores, with the most pronounced differences seen in the Impacts domain (*Figure 2*). Differences in ATTR-QOL ‘Emotional Well-being’ scores were not observed across 6-MWT groups.

Similarly, continuous ATTR-QOL scores were negatively correlated with SPPB overall scores. When patients were classified as having higher (*N* = 89) or lower (*N* = 49) functionality based on an SPPB score cut-off of 10 points, ATTR-QOL demonstrated good discriminant validity (see Supplementary material online, *Figure S3*). Patients with lower functionality had higher ATTR-QOL Symptom and Impact domain scores, indicating greater impairment by the condition. As with the 6-MWT findings, differences in ATTR-QOL Emotional Well-being scores were not observed across groups.

### ATTR-QOL and other PROs

**Figure 3:**
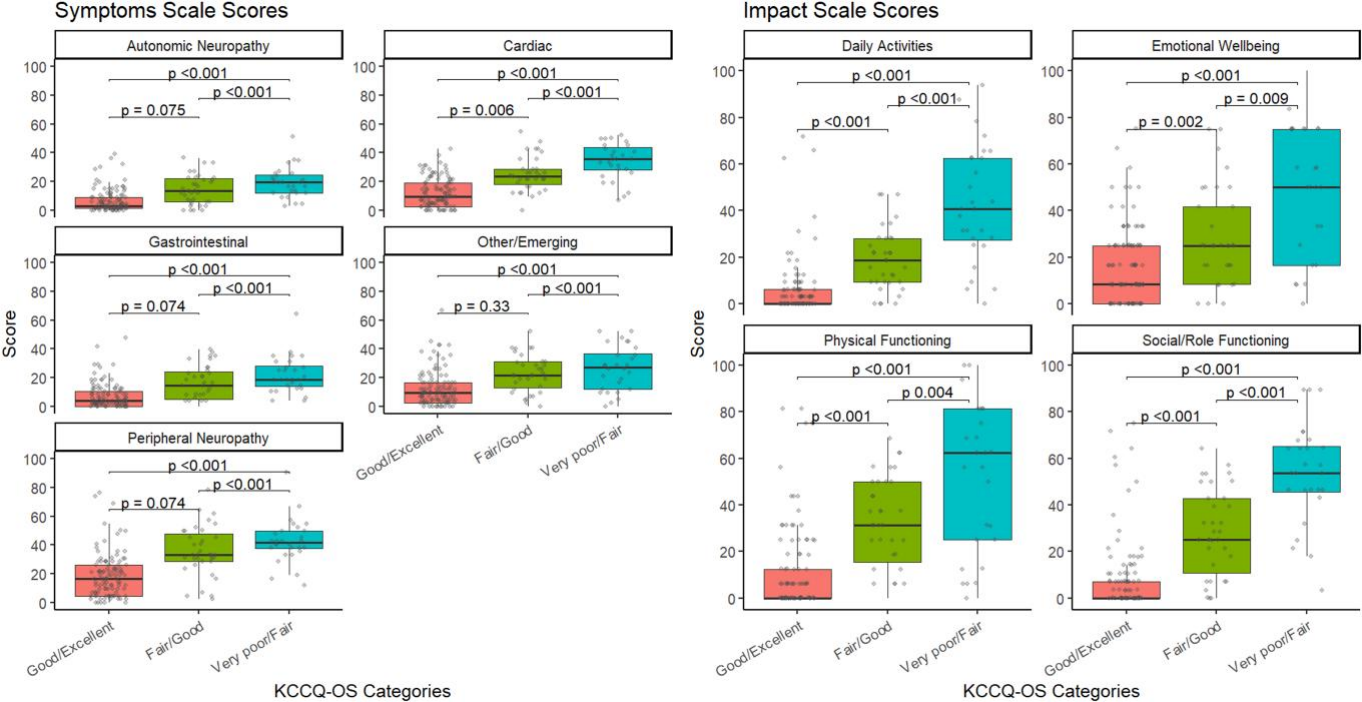
Boxplots of ATTR-QOL symptom and impact scale scores by KCCQ-OS groups: good to excellent (76–100), fair to good (51–75), very poor to fair (0–50). Higher ATTR-QOL scores indicate a greater disease burden. ATTR-QOL, Transthyretin Amyloidosis—Quality of Life Questionnaire; KCCQ-OS, Kansas City Cardiomyopathy Questionnaire Overall Score, ns, non-significant.

Finally, we examined the relationship between ATTR-QOL and other PROs, including KCCQ, SF-36v2, and COMPASS-31. Regarding KCCQ, ATTR-QOL showed a strong correlation with KCCQ physical limitation, social limitation, quality of life, and overall scores, as detailed in *Table 3*. ATTR-QOL also showed good discriminatory ability across KCCQ-OS categories (*Figure 3*), with lower KCCQ-OS scores being associated with higher ATTR-QOL scores. For SF-36v2, ATTR-QOL correlated well with SF-36v2 Physical and Mental Component Summary scores, as well as other subdomains (*Table 3*). When patients were grouped based on self-reported health status as excellent/very good (*N* = 59), good (*N* = 60), or fair/poor (*N* = 55), ATTR-QOL scores increased with worsening health status (see Supplementary material online, *Figure S4*). The ATTR-QOL demonstrated consistent correlations with COMPASS-31 overall scores, a measure of autonomic symptoms severity (*Table 3*). Overall, ATTR-QOL showed good convergent validity with established PROs and strong discriminatory ability between known groups based on other PROs.

## Discussion

This is the first study to validate the use of ATTR-QOL in clinical settings. Importantly, the present results demonstrate that ATTR-QOL: (i) is applicable in clinical settings, (ii) exhibits good convergent validity with clinical parameters, functionality, and QoL, and (iii) demonstrates known-groups discriminant validity.

The ATTR-QOL was developed as a disease-specific PRO through expert consensus, incorporating extensive patient feedback.^10-12^ Initial results from online surveys showed good performance of ATTR-QOL and association with established PROs^10-12^ Initial evaluations of the ATTR-QOL's psychometric properties indicate that the measure has good convergent validity when assessed against other established patient-reported outcomes (PROs) as criterion measures.^10-12^ Our study is the first to assess its use in a real-world clinical setting. Patients attending regular visits at our centre completed the questionnaire alongside their routine clinical assessments, significantly reducing the possibility of selection bias stemming from voluntary online surveys. This study provides the first evidence linking ATTR-QOL scores with key clinical parameters such as disease phenotype, NYHA class, biomarkers, and disease stage by Columbia and NAC scores. Our results confirm that ATTR-QOL reflects disease severity and effectively distinguishes between different severities levels, supporting its potential clinical applicability. An important consideration when evaluating its applicability in clinical settings is the time required to complete the survey. In our cohort, the median completion time was 18.5 min (range: 9.9–32.0 min). Although this duration may appear longer compared with other patient-reported outcomes (PROs), it is important to note that this instrument consolidates multiple assessments into a single questionnaire, eliminating the need for administering separate PROs.^23-25^ Collectively, this study extends the value of administering ATTR-QOL by offering unique insights and confirming associations with known clinical markers.

ATTR is a multisystemic disease that affects various aspects of life, manifesting as HF, neuropathy, and limiting physical function. Recent epidemiologic data from different countries suggest a changing pattern in phenotype, with increasing diagnosis of patients with mixed phenotype.^26-29^ Considering this, accurately capturing the patient experience requires a tool that reflects both the broad range of symptoms and their impact on daily life. While clinical trials have traditionally used PROs like KCCQ and Norfolk-QOL-Diabetic Neuropathy, these tools focus on specific symptoms and may overlook the full disease burden.^30^ The ATTR-QOL addresses this gap by providing a unified assessment of all symptoms and impacts relevant to patients with ATTR. Although only a small number of patients had a neuropathy-predominant phenotype, 48 patients presented with a mixed phenotype, and the ATTR-QOL was able to capture the full spectrum of disease burden in these patients. Our data show that the ATTR-QOL correlates well with other established PROs and effectively captures the physical symptoms related to heart failure and neuropathy, as well as other manifestations of the disease. Importantly, the ATTR-QOL separately evaluates the disease’s impact across four key domains, offering greater insight into how the disease affects daily life and helping to identify patient needs. Notably, the Symptom and Impact scores from ATTR-QOL more accurately reflect disease burden compared with general health or system specific PROs, improving the understanding of disease impact on patients. The ability to collect this comprehensive information through a single, unified questionnaire could provide more insight into the patient experience.

Our cohort showed lower numeric scores across all ATTR-QOL Symptom and Impact domains compared with previously published data.^11,12^ This likely reflects the fact that our population consisted of patients with relatively early-stage disease, as confirmed by biomarkers and NAC or Columbia staging. Previous evaluations of the ATTR-QOL lacked clinical data, making direct cross-study comparisons difficult.^11,12^ Our cohort represents a more contemporary population, reflecting the changing ATTR landscape, where earlier diagnosis leads to the identification of patients at earlier disease stages and timely initiation of DMT. Despite the lower scores, ATTR-QOL effectively distinguished between known patient groups and demonstrated strong convergent validity, confirming its value even among patients with early disease.

## Limitations

This study carries certain limitations. First, it is a single-centre, cross-sectional study, stemming from a dedicated Cardiac Amyloidosis centre, which could limit the generalizability of our findings. Additionally, the cohort included mostly older, male, wtATTR patients, which may not be reflective of the full spectrum of ATTR presentation and therefore need validation in broader populations, including more ATTR-PN or mixed phenotype patients. Second, the cohort consisted predominantly of patients with relatively early-stage disease, as indicated by biomarkers and NAC or Columbia staging. Further studies in patients with more advanced disease are needed to assess the performance of ATTR-QOL in these patients. Third, the cross-sectional design prevents assessment of changes in ATTR-QOL scores over time. Prospective data are needed to evaluate how responses to treatment and disease progression affect ATTR-QOL scores, as well as its prognostic utility. Fourth, potential selection bias may exist, as patients who agreed to participate may differ from those who declined, potentially influencing the results. Additionally, data regarding social determinants of health and diagnosis of depression, which could influence the Emotional domain, is not available in the present cohort. Finally, while ATTR-QOL showed good performance in discriminating known groups, considering the single-centre origin of the cohort and the missing data in disease staging in some cases, further validation in larger, more diverse cohorts is necessary.

## Conclusions

This study provides the first clinical validation of ATTR-QOL, demonstrating its ability to capture disease burden and distinguish between different disease severity groups in a contemporary population on DMT. However, prospective studies are needed to evaluate how ATTR-QOL scores evolve with disease progression and to evaluate its prognostic implications, which remain unknown.

## Supplementary Material

qcag015_Supplementary_Data

## Data Availability

Data will be available upon reasonable request to the authors.
