# Supplementary material for: First clinical experience with the Transthyretin Amyloidosis-Quality of Life questionnaire (ATTR-QoL): associations with clinical characteristics and established patient-reported outcomes
Source: Eur Heart J Qual Care Clin Outcomes. 2026 Feb 12;12(5):743–52. doi: 10.1093/ehjqcco/qcag015 (PMC13457938; doi:10.1093/ehjqcco/qcag015)
Supplement: Supplementary file 1 [file qcag015_supplementary_data.docx]

**Supplementary Methodology**

**STROBE Statement for the present study.**

|  | Item No. | Recommendation | Page  No. | Relevant text from manuscript |
| --- | --- | --- | --- | --- |
| **Title and abstract** | 1 | (*a*) Indicate the study’s design with a commonly used term in the title or the abstract | 2 | Line 32 |
|  |  | (*b*) Provide in the abstract an informative and balanced summary of what was done and what was found | 2 | Lines 32-45 |
| Introduction | | | |  |
| Background/rationale | 2 | Explain the scientific background and rationale for the investigation being reported | 3,4 | Lines 49-79 |
| Objectives | 3 | State specific objectives, including any prespecified hypotheses | 4 | Lines79-81 |
| Methods | | | |  |
| Study design | 4 | Present key elements of study design early in the paper | 4-7 | Lines 82-172 |
| Setting | 5 | Describe the setting, locations, and relevant dates, including periods of recruitment, exposure, follow-up, and data collection | 4 | Lines 82-100 |
| Participants | 6 | (*a*) *Cohort study*—Give the eligibility criteria, and the sources and methods of selection of participants. Describe methods of follow-up  *Case-control study*—Give the eligibility criteria, and the sources and methods of case ascertainment and control selection. Give the rationale for the choice of cases and controls  *Cross-sectional study*—Give the eligibility criteria, and the sources and methods of selection of participants | 4 | Lines 82-100 |
|  |  | (*b*) *Cohort study*—For matched studies, give matching criteria and number of exposed and unexposed  *Case-control study*—For matched studies, give matching criteria and the number of controls per case |  |  |
| Variables | 7 | Clearly define all outcomes, exposures, predictors, potential confounders, and effect modifiers. Give diagnostic criteria, if applicable | 4 | Lines 82-100 |
| Data sources/ measurement | 8* | For each variable of interest, give sources of data and details of methods of assessment (measurement). Describe comparability of assessment methods if there is more than one group | 4 | Lines 91-160 |
| Bias | 9 | Describe any efforts to address potential sources of bias | N/A | N/A |
| Study size | 10 | Explain how the study size was arrived at | 4 | Lines 83-90 |

Continued on next page

| Quantitative variables | 11 | Explain how quantitative variables were handled in the analyses. If applicable, describe which groupings were chosen and why | 6,7 | Lines 142-172 |
| --- | --- | --- | --- | --- |
| Statistical methods | 12 | (*a*) Describe all statistical methods, including those used to control for confounding | 6,7 | Lines 161-172 |
|  |  | (*b*) Describe any methods used to examine subgroups and interactions | 7 | Lines 165-170 |
|  |  | (*c*) Explain how missing data were addressed | 7 | 162-172 |
|  |  | (*d*) *Cohort study*—If applicable, explain how loss to follow-up was addressed  *Case-control study*—If applicable, explain how matching of cases and controls was addressed  *Cross-sectional study*—If applicable, describe analytical methods taking account of sampling strategy | N/A | N/A |
|  |  | (*e*) Describe any sensitivity analyses | N/A | N/A |
| Results | | | | |
| Participants | 13* | (a) Report numbers of individuals at each stage of study—eg numbers potentially eligible, examined for eligibility, confirmed eligible, included in the study, completing follow-up, and analysed | N/A | N/A |
|  |  | (b) Give reasons for non-participation at each stage | N/A | N/A |
|  |  | (c) Consider use of a flow diagram | N/A | N/A |
| Descriptive data | 14* | (a) Give characteristics of study participants (eg demographic, clinical, social) and information on exposures and potential confounders | 7 | Lines 174-184 |
|  |  | (b) Indicate number of participants with missing data for each variable of interest | 14-16 | Table 1 |
|  |  | (c) *Cohort study*—Summarise follow-up time (eg, average and total amount) | N/A | N/A |
| Outcome data | 15* | *Cohort study*—Report numbers of outcome events or summary measures over time |  |  |
|  |  | *Case-control study—*Report numbers in each exposure category, or summary measures of exposure |  |  |
|  |  | *Cross-sectional study—*Report numbers of outcome events or summary measures | 7-9 | Lines 175-223 |
| Main results | 16 | (*a*) Give unadjusted estimates and, if applicable, confounder-adjusted estimates and their precision (eg, 95% confidence interval). Make clear which confounders were adjusted for and why they were included | N/A | N/A |
|  |  | (*b*) Report category boundaries when continuous variables were categorized | 8 | Lines 199-223 |
|  |  | (*c*) If relevant, consider translating estimates of relative risk into absolute risk for a meaningful time period | N/A | N/A |

Continued on next page

| Other analyses | 17 | Report other analyses done—eg analyses of subgroups and interactions, and sensitivity analyses | 7-9 | Lines 186-223 |
| --- | --- | --- | --- | --- |
| Discussion | | | | |
| Key results | 18 | Summarise key results with reference to study objectives | 9 | Lines 225-228 |
| Limitations | 19 | Discuss limitations of the study, taking into account sources of potential bias or imprecision. Discuss both direction and magnitude of any potential bias | 10-11 | Lines 274-286 |
| Interpretation | 20 | Give a cautious overall interpretation of results considering objectives, limitations, multiplicity of analyses, results from similar studies, and other relevant evidence | 9-11 | Lines 229-271 |
| Generalisability | 21 | Discuss the generalisability (external validity) of the study results | 9-11 | Lines 263-294 |
| Other information | |  | | |
| Funding | 22 | Give the source of funding and the role of the funders for the present study and, if applicable, for the original study on which the present article is based | 11 | Line 296 |

*Give information separately for cases and controls in case-control studies and, if applicable, for exposed and unexposed groups in cohort and cross-sectional studies.

**Note:** An Explanation and Elaboration article discusses each checklist item and gives methodological background and published examples of transparent reporting. The STROBE checklist is best used in conjunction with this article (freely available on the Web sites of PLoS Medicine at http://www.plosmedicine.org/, Annals of Internal Medicine at http://www.annals.org/, and Epidemiology at http://www.epidem.com/). Information on the STROBE Initiative is available at www.strobe-statement.org.

**Supplementary Table 1.** Demographics and baseline characteristics of the patients included in the present analysis vs the patients excluded. Abbreviations: ATTR, transthyretin amyloidosis; eGRR= estimated glomerular filtration rate, NtprobBNP= N-terminal pro B natriuretic peptide, hs-TnT= high sensitivity troponin T, ACEi= angiotensin converting enzyme inhibitors, MCRA= mineralocorticoid receptor antagonist, SGLT2i= sodium glucose co-transporter inhibitor, NAC, National Amyloidosis Center; NYHA, New York Heart Association, HF =heart failure, KCCQ= Kansas city cardiomyopathy questionnaire.

| **Characteristic** | **No ATTR-QOL (n=140)** | | **ATTR-QOL (n=194)** | | **p-value** |
| --- | --- | --- | --- | --- | --- |
|  | **N** |  | **N** |  |  |
| **Demographic** |  |  |  |  |  |
| Age ̶ yr | 140 | 79 (71.5, 87) | 194 | 80 (72, 85) | 0.62 |
| Sex ̶ no. (%) |  |  |  |  | ***0.046*** |
| Male | 140 | 107 (76.4) | 194 | 165 (85.1) |  |
| Female | 140 | 33 (23.6) | 194 | 29 (15) |  |
| Race ̶ no. (%) |  |  |  |  | 0.06 |
| White | 139 | 93 (66.9) | 194 | 148 (76.3) |  |
| Black | 139 | 30 (21.6) | 194 | 22 (11.3) |  |
| Other | 139 | 1 (0.7) | 194 | 4 (2.1) |  |
| Unknown or not reported | 139 | 15 (10.8) | 194 | 20 (10.3) |  |
| Ethnicity ̶ no. (%) |  |  |  |  | >0.99 |
| Hispanic | 139 | 5 (3.6) | 193 | 7 (3.6) |  |
| Non-Hispanic | 139 | 119 (85.6) | 193 | 165 (85.5) |  |
| Unknown or not reported | 139 | 15 (10.8) | 193 | 21 (10.9) |  |
| **Past medical history** |  |  |  |  |  |
| Atrial fibrillation | 127 | 57 (44.9) | 194 | 87 (44.9) | >0.99 |
| Coronary artery disease | 124 | 36 (29) | 194 | 52 (26.8) | 0.66 |
| Diabetes mellitus | 127 | 12 (9.5) | 194 | 15 (7.7) | 0.59 |
| Chronic kidney disease | 120 | 41 (34.2) | 191 | 36 (18.9) | ***0.002*** |
| Peripheral Neuropathy | 110 | 25 (22.7) | 190 | 43 (22.6) | 0.98 |
| Autonomic neuropathy | 106 | 4 (3.8) | 187 | 9 (4.8) | 0.78 |
| Carpal tunnel syndrome | 107 | 45 (42.1) | 185 | 96 (51.9) | 0.11 |
| Spinal stenosis | 107 | 16 (15) | 188 | 71 (37.8) | ***<0.001*** |
| **Laboratory** |  |  |  |  |  |
| eGFR ̶ mL/min/1.73m^2^ | 132 | 58.5 (47.7, 75) | 186 | 59.5 (47.2, 72.1) | 0.79 |
| NTproBNP ̶ pg/mL | 131 | 1112 (471.8, 3085) | 175 | 1108 (360, 2608) | 0.74 |
| hs-TnT ̶ ng/L | 123 | 40 (18, 56) | 156 | 38.5 (23.5, 56) | 0.43 |
| **Medications** |  |  |  |  |  |
| Loop diuretic ̶ no. (%) | 109 | 59 (54.1) | 190 | 94 (49.5) | 0.44 |
| Beta-blocker ̶ no. (%) | 124 | 25 (20.2) | 193 | 55 (28.5) | 0.1 |
| ACE-I or ARB ̶ no. (%) | 124 | 23 (18.6) | 193 | 31 (16.1) | 0.57 |
| MCRA ̶ no. (%) | 124 | 28 (22.6) | 193 | 86 (44.6) | ***<0.001*** |
| SGLT2i ̶ no. (%) | 124 | 41 (33.1) | 193 | 88 (45.6) | ***0.03*** |
| **Disease modifying therapy** |  |  |  |  |  |
| None | 140 | 22 (15.7) | 193 | 0 (0) | ***<0.001*** |
| Stabilizer only | 140 | 96 (68.6) | 193 | 157 (80.9) |  |
| Silencer only | 140 | 5 (3.6) | 193 | 9 (4.6) |  |
| Stabilizer and silencer | 140 | 17 (12.1) | 193 | 28 (14.4) |  |
| **HF staging and ATTR-CM risk score** |  |  |  |  |  |
| NYHA classification ̶ no. (%) |  |  |  |  | ***0.01*** |
| Class I | 129 | 27 (20.9) | 189 | 52 (26.8) |  |
| Class II | 129 | 60 (46.5) | 189 | 100 (51.6) |  |
| Class III | 129 | 38 (29.5) | 189 | 42 (21.6%) |  |
| Class IV | 129 | 4 (3.1) | 189 | 0 (0) |  |
| NAC stage |  |  |  |  | 0.71 |
| Stage I | 130 | 83 (63.9) | 171 | 114 (66.7) |  |
| Stage II | 130 | 31 (23.9) | 171 | 41 (24) |  |
| Stage III | 130 | 16 (12.3) | 171 | 16 (9.4) |  |
| **KCCQ** |  |  |  |  |  |
| Clinical summary | 103 | 81.6 (59.4, 93.3) | 177 | 83.4 (67.8, 98) | 0.23 |
| Overall summary | 103 | 80.8 (58.4, 94.9) | 177 | 82.3 (63.5, 95.7) | 0.43 |

**Supplementary Table 2.** Score Distributions of ATTR-QOL Symptom and Impact Scale Scores. Abbreviations: ATTR-QOL, Transthyretin Amyloidosis Quality of Life Questionnaire; SD, Standard Deviation

| **Scale** | **N** | **Mean (SD)** | **Median** | **Observed Range** | **Possible Range** | **% Floor** | **% Ceiling** |
| --- | --- | --- | --- | --- | --- | --- | --- |
| ***ATTR-QOL Symptom Scale Scores*** | | | | | | | |
| **Cardiac** | 192 | 17.7 (13.7) | 15.5 | 0.0 - 54.8 | 0.0 - 100.0 | 12.5% | 0.0% |
| **Peripheral Neuropathy** | 192 | 25.1 (18.8) | 21.4 | 0.0 - 90.5 | 0.0 - 100.0 | 6.8% | 0.0% |
| **Autonomic Neuropathy** | 192 | 10.1 (10.1) | 6.1 | 0.0 - 51.5 | 0.0 - 100.0 | 16.1% | 0.0% |
| **Gastrointestinal** | 194 | 11.2 (11.7) | 8.3 | 0.0 - 64.6 | 0.0 - 100.0 | 20.3% | 0.0% |
| **Other/Emerging** | 194 | 16.3 (14.0) | 11.9 | 0.0 - 66.7 | 0.0 - 100.0 | 10.4% | 0.0% |
| ***ATTR-QOL Impact Scale Scores*** | | | | | | | |
| **Daily Activities** | 194 | 14.3 (20.2) | 6.3 | 0.0 - 93.8 | 0.0 - 100.0 | 38.1% | 0.0% |
| **Physical Functioning** | 194 | 22.8 (27.7) | 6.3 | 0.0 - 100.0 | 0.0 - 100.0 | 37.6% | 2.1% |
| **Social/Role Functioning** | 194 | 18.5 (23.5) | 7.1 | 0.0 - 89.3 | 0.0 - 100.0 | 41.2% | 0.0% |
| **Emotional Wellbeing** | 194 | 20.7 (23.0) | 16.7 | 0.0 - 100.0 | 0.0 - 100.0 | 33.5% | 0.5% |

**Supplementary Figure 1.** Boxplots of ATTR-QOL symptom and impact scale scores by NYHA class. Higher ATTR-QOL scores indicate a greater disease burden. Abbreviations: ATTR-QOL= Transthyretin Amyloidosis – Quality of Life Questionnaire; NYHA= New York Heart Association, ns= non-significant

**Supplementary Figure 2.** Boxplots of ATTR-QOL symptom and impact scale scores by Columbia stage. Higher ATTR-QOL scores indicate a greater disease burden. Abbreviations: ATTR-QOL= Transthyretin Amyloidosis – Quality of Life Questionnaire, ns= non-significant

**Supplementary Figure 3.** Boxplots of ATTR-QOL symptom and impact scale scores by SPPB categories (≥10 points). Higher ATTR-QOL scores indicate a greater disease burden. Abbreviations: ATTR-QOL= Transthyretin Amyloidosis – Quality of Life Questionnaire; SPPB= Short Physical Performance Battery Test. *p<0.05, **p<0.01, ***p<0.001, ns= non-significant

**Supplementary Figure 4.** Boxplots of ATTR-QOL symptom and impact scale scores by SF-36v2 general health categories. Higher ATTR-QOL scores indicate a greater disease burden. Abbreviations: ATTR-QOL= Transthyretin Amyloidosis – Quality of Life Questionnaire. *p<0.05, **p<0.01, ***p<0.001, ns= non-significant
